# Effectiveness and safety of hydrogen inhalation as an adjunct treatment in Chinese type 2 diabetes patients: A retrospective, observational, double-arm, real-life clinical study

**DOI:** 10.3389/fendo.2022.1114221

**Published:** 2023-01-18

**Authors:** Ziyi Zhao, Hongxiang Ji, Yunsheng Zhao, Zeyu Liu, Ruitao Sun, Yuquan Li, Tongshang Ni

**Affiliations:** ^1^ School of Clinical Medicine, Department of Medicine, Qingdao University, Qingdao, China; ^2^ College of Traditional Chinese Medicine, Shandong University of Traditional Chinese Medicine, Jinan, China; ^3^ Department of Endocrinology, Qingdao Hospital of Traditional Chinese Medicine (Qingdao Hiser Hospital), Qingdao, China; ^4^ Center of Integrated Traditional Chinese and Western Medicine, Department of Medicine, Qingdao University, Qingdao, China

**Keywords:** type 2 diabetes, observational study, hydrogen inhalation, glycemic control, real world study

## Abstract

**Aim:**

To analyze the effectiveness and safety of hydrogen inhalation (HI) therapy as an adjunct treatment in Chinese type 2 diabetes mellitus (T2DM) patients in a real-life clinical setting.

**Methods:**

This observational, non-interventional, retrospective, double-arm, 6-month clinical study included T2DM patients receiving conventional anti-diabetes medication with or without HI initiation from 2018 to 2021. Patients were assigned to the HI group or non-HI group (control group) after 1:1 propensity score matching (PSM). The mean change in glycated hemoglobin (HbA1c) after 6 months in different groups was evaluated primarily. The secondary outcome was composed of the mean change of fasting plasma glucose (FPG), weight, lipid profile, and homeostasis model assessment. Logistics regression was performed to evaluate the likelihood of reaching different HbA1c levels after 6-month treatment between the groups. Adverse event (AE) was also evaluated in patients of both groups.

**Results:**

In total, 1088 patients were selected into the analysis. Compared to the control group, subjects in HI group maintained greater improvement in the level of HbA1c (-0.94% vs -0.46%), FPG (-22.7 mg/dL vs -11.7 mg/dL), total cholesterol (-12.9 mg/dL vs -4.4 mg/dL), HOMA-IR (-0.76 vs -0.17) and HOMA-β (8.2% vs 1.98%) with all p< 0.001 post the treatment. Logistics regression revealed that the likelihood of reaching HbA1c< 7%, ≥ 7% to< 8% and > 1% reduction at the follow-up period was higher in the HI group, while patients in the control group were more likely to attain HbA1c ≥ 9%. Patients in HI group was observed a lower incidence of several AEs including hypoglycemia (2.0% vs 6.8%), vomiting (2.6% vs 7.4%), constipation (1.7% vs 4.4%) and giddiness (3.3% vs 6.3%) with significance in comparison to the control group.

**Conclusion:**

HI as an adjunct therapy ameliorates glycemic control, lipid metabolism, insulin resistance and AE incidence of T2DM patients after 6-month treatment, presenting a noteworthy inspiration to existing clinical diabetic treatment.

## 1 Introduction

Diabetes is one of the most common chronic metabolic diseases with global morbidity of 9.3% in 2019 and expected 10.9% in 2045 (1), while Asian countries account for more than 60% of the diabetic population worldwide (2). China owns the largest number of diabetic patients in the world which is up to 109 million (3). T2DM contributes to more than 91% of all diabetes cases, which is estimated to cost more than 2 billion USD after a decade, while most of the costs are brought by complications which could be largely avoided by reaching and remaining glycemic target (4–6).

The current therapeutic strategy for T2DM mainly includes lifestyle amelioration and medication. If the patient fails to gain adequate glycemic control *via* lifestyle intervention composed of changes in diet and exercise (7), hypoglycemic medication initiation is recommended. As diabetes processes, Intensification treatment with multiple drugs will be utilized if required, which includes dual combination therapy, triple combination therapy or the addition of insulin (8).

However, the oral antidiabetic drug often results in insulin resistance or adverse event, leading to low efficacy (9). Optimal glycemic control is hard to obtain after insulin usage on account of the increasing risk of weight gain and hypoglycemia (10). Besides, the HbA1c target of< 7% is maintained by no more than half of the patients taking basal insulin in a meta-analysis (11). Although the American Diabetes Association (ADA) and the European Association for the Study of Diabetes (EASD) recommended the glucagon-like peptide 1 receptor agonist as the first choice of injectable medication for individuals with T2DM, patients’ compliance in the real-world study (RWS) is more than low, resulting in a non-negligible difference between RWS and randomized controlled trial (RCT) (5, 6, 12, 13). Despite antidiabetic medicine updating in the past few years, no more than half of Chinese diabetic patients have the capability to gain adequate glycemic control (14). Furthermore, comprehensive and effective therapy for patients with multiple metabolic disorders is especially demanded (15).

Oxidative stress is defined as an imbalance between the production of reactive oxygen species (ROS) and the activity of the antioxidant defense system (16), which is widely concerned with multiple diseases including ischemia-reperfusion injury and inflammatory and neurological disease (17). Physiological ROS plays an important role in the maintenance and regulation of various physiological processes while redundant production of ROS would result in damage to proteins, DNA and lipids (18, 19). The development of metabolic disorders in diabetes are related to oxidative stress and inflammation (20). Thus, quenching or lowering ROS may be a potential antidiabetic means. Previous study suggested that antioxidant vitamins have a positive impact on glycemic control in T2DM patients (21). However, vitamin antioxidants application is associated with several adverse effects including mortality increase and heart failure (22, 23).

Hydrogen (H2) is a newly developed antioxidant which could diffuse across cytomembrane easily and selectively eliminate cytotoxic ROS without influencing ROS acting as parts of physiological function (24, 25). Furthermore, molecular hydrogen has been proved of a quantity of preventive and therapeutic effects in human and animal disease models, which include ischemia–reperfusion injuries (26, 27), neurodegeneration (28, 29), cardiovascular diseases (30, 31), metabolic syndrome (32), inflammation (33, 34) and cancer (35, 36). Recently, there are numerous reports about the prominent effect of molecular hydrogen in both T2DM patients and diabetic animal models (37–41). Previous RCT has already revealed the fact that the high-concentration hydrogen-rich water could attenuate the HbA1c and FPG levels of 60 patients with metabolic syndrome in 24-week treatment (42). Although RCT is the golden standard for evidence-based medicine, RWS displays clinical experience extensively and the diversity of patients’ distribution, offering a better insight into the real-life medical situation.

There are plenty of molecular hydrogen ingestion methods, including oral ingestion by drinking hydrogen water, hydrogen-saline injection, direct incorporation of molecular hydrogen by diffusion, maternal intake of hydrogen and hydrogen gas inhalation (43). With the progressive popularization of hydrogen medicine, the antidiabetic effect of molecular hydrogen is extensively acknowledged. While the utilization of medicinal gases as a therapeutic strategy is gaining ever-growing attention, hydrogen inhalation (HI) is widely accepted by the Chinese public, with a substantial amount of manufactory producing hydrogen generation machines (44, 45). An increasing number of Chinese diabetic patients are taking HI by applying the hydrogen-producing machine as a means of maintaining adequate glucose control. However, no research has focused on the effect of T2DM patients utilizing HI ever before to our knowledge.

Therefore, this retrospective study aims at revealing the effectiveness and safety of HI as an adjunct treatment in Chinese patients with type 2 diabetes in a real-world setting, adding more data to the evidence base of T2DM patients receiving HI treatment as explorational research.

## 2 Material and methods

### 2.1 Study design and patient population

This is a non-interventional, retrospective, multicenter, observational, double-arm study to analyze the real-world effectiveness and safety of HI as an adjunct treatment in Chinese patients with T2DM. The data were selected from electronic medical records in the multi-institutional health records database, which is composed of individual demographic characteristics, medical procedures, diagnoses (International Classification of Diseases, 10th Revision) and medicinal prescriptions from multiple hospitals and health examination centers in Tsingtao, China. All the eligible patients were anonymous by utilizing special study numbers.

Type 2 diabetes patients who kept conventional hypoglycemic treatment for at least 6 months with or without HI initiation between January 1, 2018 and December 31, 2021 were considered eligible for this study. Subjects were assigned to the HI group or non-HI group (control group) depending on previous treatment details. In the HI group, individuals without former molecular hydrogen treatment initiated and kept hydrogen therapy in the specific hydrogen therapy departments where the hydrogen gas was generated by the hydrogen-producing machine (HZS-2700A, Qingdao Haizhisheng Corp.,LTD, Tsingtao, China) which provided 100% hydrogen gas at the speed of 3000 ml/h by water electrolysis. The index date was defined as the time when HI was initiated or hypoglycemic medication was applied. The follow-up was defined as 6 ±; 1 months after the index date while 1 month before and after the index date was defined as the baseline.

Patients were included in the study in accordance with the following criteria: (1) Chinese adults (≥ 18 years of age) of both sexes. (2) diagnosed with T2DM. (3) at least one laboratory value in both periods of baseline and follow-up. (4) at least 25 hours of HI therapy per week or at least one hypoglycemic prescription at the baseline throughout the study. Meanwhile, the exclusion criteria are (1) type 1 or any other specific diabetes (e.g. gestational, secondary, steroid). (2) maintaining concurrent severe medical diseases or severe mental conditions. (3) pregnant or lactating. (4) participation in other studies simultaneously.

### 2.2 Data collection and assessments

Baseline characteristics included gender, age, HbA1c, diabetes duration, body mass index (BMI), lipid profile (triglyceride, total cholesterol, low-density lipoprotein and high-density lipoprotein), diabetes complications (i.e., diabetic retinopathy, neuropathy, nephropathy), cardiovascular diseases (i.e., angina pectoris, myocardial infarction, heart failure), antihyperglycemic treatment (i.e., biguanides, thiazolidinediones, insulin) and antihypertensive therapy (i.e., diuretics, beta-blockers, calcium channel blockers). HbA1c, FPG, lipid profile, homeostasis model assessment of insulin resistance (HOMA-IR), homeostasis model assessment of β-cell function (HOMA-β) and weight were assessed at the follow-up.

HOMA-IR and HOMA-β were determined as follows:


HOMA−IR=FPG (mg/dL) × FINS (µU/L)/405

HOMA−β= 360×FINS (µU/L)/(FPG (mg/dL) – 63) (%)

Safety was evaluated by recording hypoglycemia, adverse event (AE) and serious adverse event (SAE) during the study. All the AEs were extracted from the medical records documented by their attending physicians.

The change of HbA1c level from baseline to follow-up was evaluated primarily. The secondary endpoint included the change in FPG, weight, HOMA-IR, HOMA-β and lipid profile after 6 months. The proportion of patients with HbA1c level of< 7%, ≥ 7% to< 8%, ≥ 8% to< 9%, ≥ 9% and > 1% reduction after the 6-month follow-up period were also analyzed.

For subjects having more than one laboratory data in the baseline and follow-up period, the values closest to the index date and 6 months post index date were used.

### 2.3 Statistical analysis

To adjust the possible selective bias and to make the cohorts more comparable and homogeneous, the PSM was applied. Depending on the baseline parameters displayed in **Table 1**, a logistic regression model was established to estimate the propensity scores of different treatment groups. The PSM was performed with a greedy nearest neighbor 1:1 matching technique and a caliper of 0.05 on the propensity score scale.

**Table 1:** Baseline characteristics of patients after propensity score matching in the study.

Variable	HI group (N = 544)	Control group (N = 544)	P-value
Gender (female)	278 (51.1%)	269 (49.4%)	0.182
Age, years	62.4 ± 8.9	63.1 ± 9.3	0.256
BMI, kg/m2	28.3 ± 3.5	28.8 ± 3.3	0.462
Diabetes duration, years	10.1 ± 3.0	9.9 ± 2.9	0.059
HbA1c, %	8.98 ± 0.85	9.03 ± 0.85	0.697
Lipid profile			
TG, mg/dL	160.0 ± 40.1	160.7 ± 41.0	0.783
TC, mg/dL	170.0 ± 37.2	169.1 ± 36.3	0.478
HDL, mg/dL	44.1 ± 7.8	44.3 ± 7.8	0.864
LDL, mg/dL	99.2 ± 15.0	97.9 ± 15.7	0.192
Diabetes complications			
Diabetic retinopathy	61 (11.2)	72 (13.2)	0.098
Diabetic neuropathy	108 (19.9)	116 (21.3)	0.752
Diabetic nephropathy	89 (16.4)	80 (14.7)	0.061
Cardiovascular diseases			
Hypertension	304 (55.9)	310 (57.0)	0.382
Hyperlipidemia	352 (64.7)	359 (66.0)	0.092
Coronary heart disease	112 (20.6)	118 (21.7)	0.216
Peripheral artery disease	12 (2.2)	10 (1.8)	0.546
Angina pectoris	127 (23.3)	125 (23.0)	0.896
Myocardial infarction	15 (2.8)	17 (3.1)	0.476
Heart failure	16 (2.9)	17 (3.1)	0.912
Dementia	2 (0.4)	3 (0.6)	0.745
Stroke	103 (18.9)	98 (18.0)	0.392
Cancer	56 (10.3)	51 (9.3)	0.298
Chronic obstructive pulmonary disease	34 (6.3)	36 (6.6)	0.465
Liver disease	9 (1.7)	8 (1.5)	0.341
Background anti-diabetes medications			
Biguanides	326 (59.9)	330 (60.7)	0.348
Sulphonylureas	168 (30.9)	162 (29.8)	0.548
Thiazolidinediones	82 (15.1)	83 (15.3)	0.786
α-glucosidase inhibitors	60 (11.0)	58 (10.7)	0.723
DPP-4 inhibitors	65 (11.9)	61 (11.2)	0.218
SGLT-2 inhibitors	38 (7.0)	39 (7.2)	0.812
GLP-1 receptor agonists	27 (5.0)	30 (5.5)	0.409
Insulin	351 (64.5)	356 (65.4)	0.293
Glinides	11 (2.0)	9 (1.7)	0.582
Other	2 (0.4)	1 (0.2)	0.801
Blood pressure–lowering medications			
Diuretics	65 (11.9)	64 (11.8)	0.908
ACE inhibitors	54 (9.9)	57 (10.5)	0.168
ARBs	243 (44.7)	246 (45.2)	0.380
Beta-blockers	136 (25.0)	132 (24.3)	0.731
Calcium channel blockers	216 (39.7)	218 (40.1)	0.490
Other	4 (0.7)	3 (0.6)	0.812

All data are presented as mean ±; SD or as number (%).

HI, hydrogen inhalation; BMI, body mass index; HbA1c, glycated hemoglobin; TG, triglycerides; TC, Total Cholesterol; HDL, high-density lipoprotein; LDL, low-density lipoprotein; DPP-4 dipeptidyl peptidase-4; SGLT-2, sodium-glucose co-transporter-2; GLP-1 glucagon-like peptide-1; ACE angiotensin-converting enzyme; ARB angiotensin-receptor blockers.

Numerical data were expressed as the mean ± standard deviation (SD). Paired t-tests were applied to measure the differences in laboratory parameters from baseline to the end of the study within the same cohort, meanwhile, the independent t-tests were to analyze the changes in laboratory indicators between different groups. The means with 95% confidence interval (CI) were used to demonstrate the changes in laboratory parameters after 6-month treatment. Ordinal data were presented as frequency (percentage) and analyzed by the χ2 test. the logistic regression model was performed to examine the proportion of patients reaching HbA1c level< 7%, ≥ 7% to< 8%, ≥ 8% to< 9%, ≥ 9% and > 1% reduction after 6 months. odds ratio (OR) and 95% CI were calculated to compare the different cohorts.

To evaluate the robustness of the study, repeated analyses had been performed in subgroups based on several baseline values, including HbA1c (stratified by< 8% and ≥ 8%), BMI (stratified by< 28 kg/m2 and ≥ 28 kg/m2), usage of hypoglycemic drugs prior to the study (stratified by< 2 and ≥ 2), as previous studies showed that distinct BMI, glycemic level and former hypoglycemic treatment failures at the baseline may lead to different anti-diabetic treatment effect (46–49).

To display statistically significant differences in HbA1c change between different treatment groups with a power of 95%, each cohort demanded the sample size of 445 patients, assuming a minimum expected reduction in HbA1c of 0.1%, SD of 0.5% and 0.01 in two-sided significant level.

Statistical analyses were carried out with SAS 9.2 (SAS Institute, Cary, NY, USA). All statistical tests were two-tailed tests and considered the p-value<0.05 as statistically significant.

## 3 Result

### 3.1 patient flow and baseline characteristics

**Figure 1:**
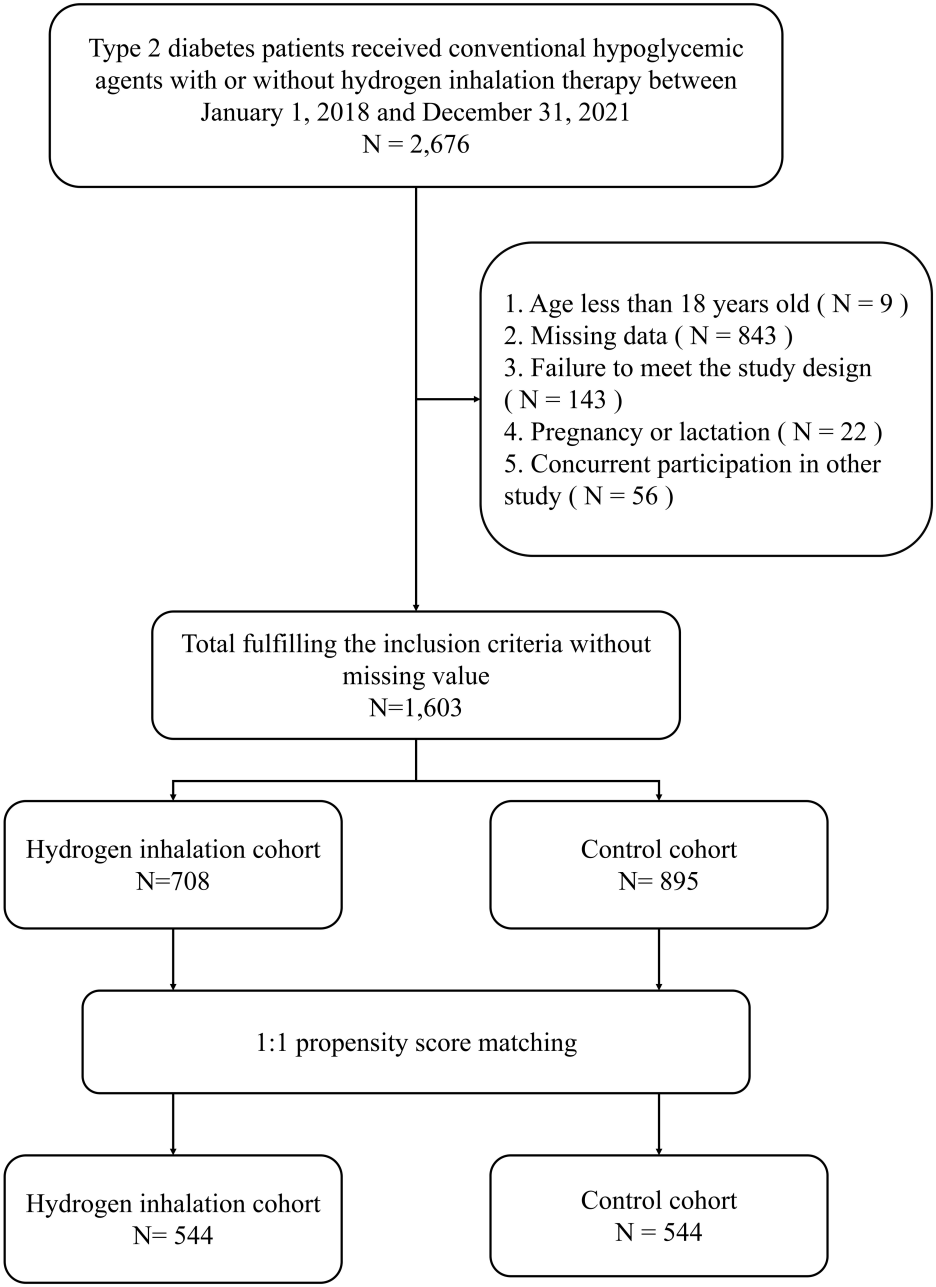
flowchart of patient selection.

Depending on the inclusion and exclusion criteria, a total number of 1,603 patients were identified, composed of 708 subjects initiating HI therapy and 895 individuals treated with conventional hypoglycemic agents.544 pairs were selected for analysis after 1:1 propensity score matching (**Figure 1**). Patient demographic and clinical characteristics at baseline were comparable between the two cohorts, including gender, age, diabetes duration, HbA1c level, lipid profile, diabetes complications, cardiovascular diseases and concomitant hypoglycemic and antihypertensive medications (all p-value > 0.05) (**Table 1**).

### 3.2 Effectiveness evaluation

**Figure 2:**
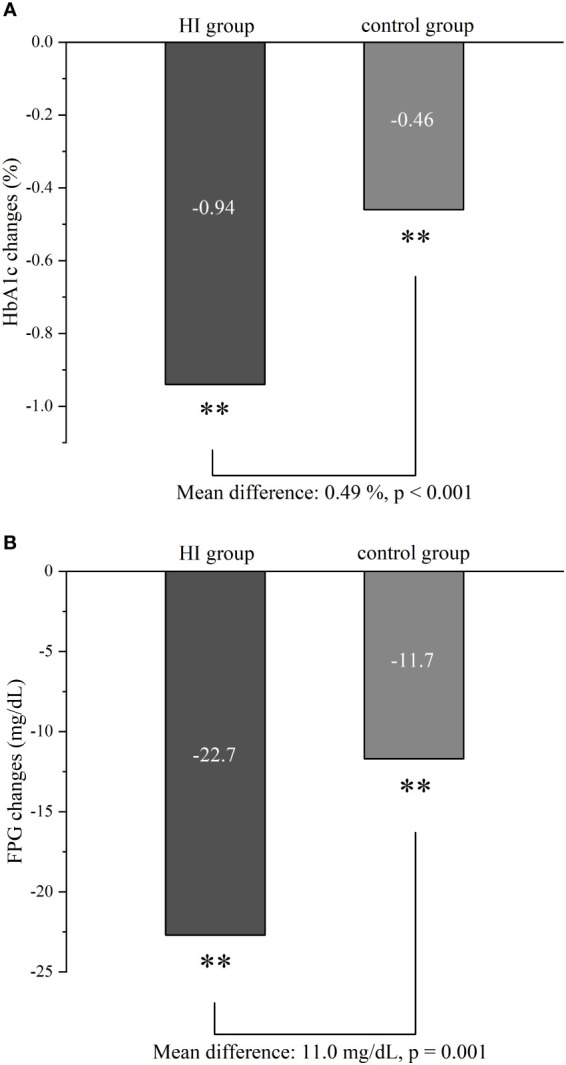
Comparison of glycemic control in HbA1c **(A)** and FPG **(B)** between HI group and control group from baseline to follow-up. **p < 0.001. Abbreviation: HI, hydrogen inhalation; HbA1c, glycated hemoglobin; FPG, fasting plasma glucose.

After 6-month treatment, both the levels of HbA1c and FPG decreased in HI group (-0.94%; 95% CI -1.04 to -0.85 and -22.7 mg/dL; 95% CI -27.0 to -18.5) and control group (-0.46%; 95% CI -0.56 to -0.35 and -11.7 mg/dL; 95% CI -16.5 to -7.0) with all p< 0.001. Meanwhile, greater reductions in HbA1c and FPG were observed in the HI group (p< 0.001 and p = 0.001 separately) (**Figure 2**).

**Figure 3:**
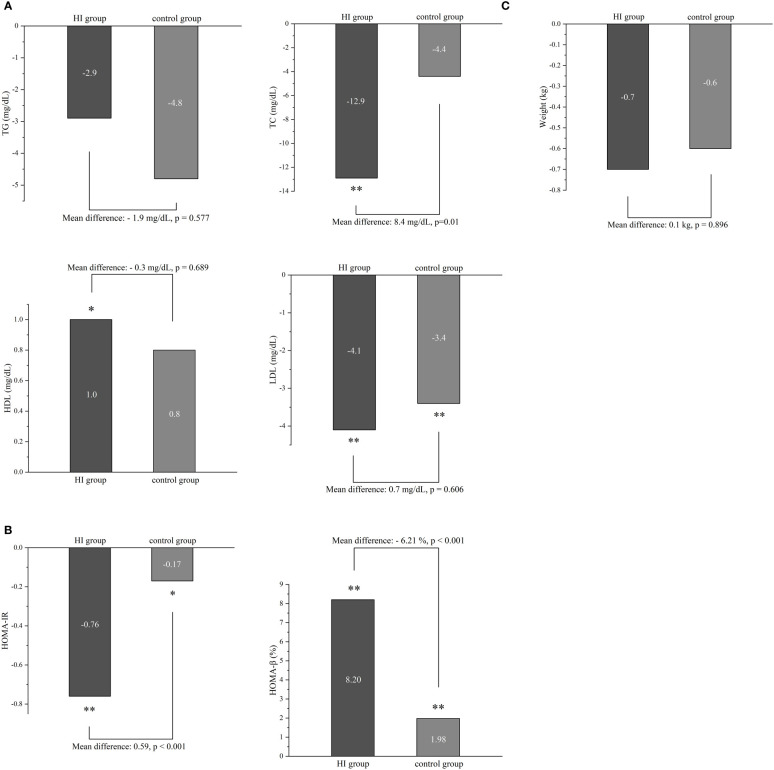
The difference in lipid profile **(A)**, insulin resistance **(B)** and body weight **(C)** between two groups during the study. *p < 0.05, **p < 0.001. Abbreviation: HI, hydrogen inhalation; TG, triglycerides; TC, Total Cholesterol; HDL, high-density lipoprotein; LDL, low-density lipoprotein; HOMA-IR, homeostasis model assessment of insulin resistance; HOMA-β, homeostasis model assessment of β-cell function.

HI therapy could lead to significant improvement in TC (-12.9 mg/dL; 95% CI -17.4 to -8.3), HDL (1.0 mg/dL; 95% CI 0.2 to 1.9) and LDL (-4.1 mg/dL; 95% CI -6.0 to -2.3). Meanwhile, it demonstrated reduction of LDL level (-3.4 mg/dL; 95% CI -5.3 to -1.6) with p< 0.001 in patients treated with conventional anti-diabetes medication. Compared to the control group, HI therapy only showed the favorable effect on TC (p=0.01) (**Figure 3A**).

There were significant improvements of both HOMA-IR and HOMA-β in HI group (-0.76; 95% CI -0.87 to -0.66 and 8.20%; 95% CI 7.28 to 9.11) and control group (-0.17; 95% CI -0.28 to -0.06 and 1.98%; 95% CI 1.09 to 2.87). fewer ameliorations were displayed in the control group of both indexes (all p< 0.001) (**Figure 3B**). Neither HI therapy nor conventional hypoglycemic agents could significantly influence body weight after 6 months (**Figure 3C**).

**Figure 4:**
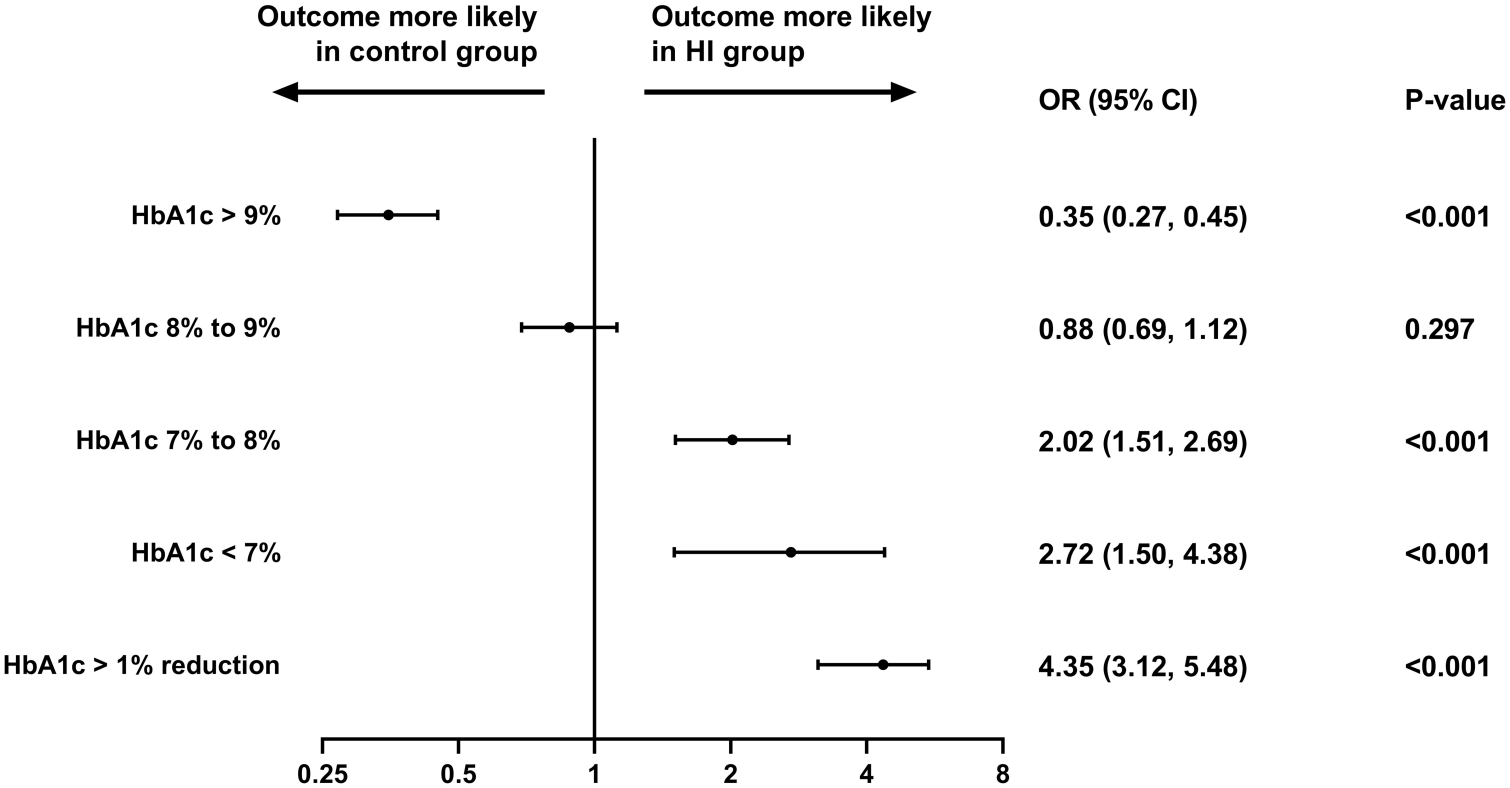
Different HbA1c level attainment at the follow-up period in the study. Abbreviation: HI, hydrogen inhalation; HbA1c, glycated hemoglobin; OR, odds ratio; CI, confidence interval.

The likelihood of reaching HbA1c< 7%, ≥ 7% to< 8% and > 1% reduction at the follow-up period was higher in HI group with OR (95% CI) of 2.72 (1.50, 4.38), 2.02 (1.51, 2.69) and 4.35 (3.12, 5.48) separately, while subjects in control group were more likely to attain HbA1c ≥ 9% (all p< 0.001). The similar possibility of having HbA1c ≥ 8% to< 9% was presented in both cohorts (**Figure 4**).

### 3.3 Safety outcome

The gastrointestinal adverse event was the most common AE both in HI group (10.9%) and control group (15.9%). Compared to control group, the HI therapy leaded to lower incidence of several AEs including hypoglycemia (2.0% vs 6.8%, p< 0.001), vomiting (2.6% vs 7.4%, p< 0.001), constipation (1.7% vs 4.4%, p = 0.008) and giddiness (3.3% vs 6.3%, p = 0.023). There was no SAE recorded throughout the whole study. The frequency of adverse event episode incidence is summarized in **Table 2**.

**Table 2:** Adverse events recorded in the HI group and control group throughout the study.

Variable	HI group (N=544)	Control group (N=544)	P-value
Hypoglycemia	11 (2.0)	37 (6.8)	<0.001
Abdominal pain	7 (1.3)	15 (2.8)	0.085
Vomiting	14 (2.6)	40 (7.4)	<0.001
Bloating	4 (0.7)	7 (1.3)	0.363
Constipation	9 (1.7)	24 (4.4)	0.008
Urinary tract infection	21 (3.9)	27(5.0)	0.376
Palpitation	18 (3.3)	12 (2.2)	0.267
Giddiness	18 (3.3)	34 (6.3)	0.023

Data are displayed as number (%).

HI, hydrogen inhalation.

## 4 Discussion

As no similar research has been carried out in this ever-growing field before, this observational, non-interventional, retrospective, double-arm, multicenter study focuses on revealing the effectiveness and safety of HI as an adjunct treatment in Chinese patients with type 2 diabetes in a real-world setting. Compared to conventional anti-diabetes medication, HI therapy is associated with greater amelioration in HbA1c, FPG, TC level and insulin resistance. Treatment with HI therapy versus control group leads to the higher likelihood of reaching HbA1c< 7%, ≥ 7% to< 8%, > 1% reduction and a lower possibility of achieving HbA1c ≥ 9% after 6 months.

Although hydrogen-water was often utilized in previous diabetic studies, low saturation of hydrogen would limit the body abortion (39). Otherwise, the comparison result which shows the effect of different hydrogen administration methods in normal rats also reveals that most serum biochemical factors alter more significantly in HI group than the hydrogen-water group, which is at least partially due to its longer period of hydrogen concentration maintenance (50, 51).

In comparison to patients treated with conventional hypoglycemic medication, significantly greater reductions of HbA1c and FPG are observed in subjects receiving HI therapy at the follow-up period. It is in accordance with the result of previous RCT revealing that hydrogen-rich water could ameliorate HbA1c and FPG level (42). However, a study applying hydrogen-rich water to 36 T2DM patients showed no significance in lowering the HbA1c concentration, which might be due to different usage of the molecular hydrogen and low sample size (41). Several previous animal researches also indicate that molecular hydrogen is associated with FPG-lowering effect (37, 38, 40). The glycemic improvement might be realized by activating phosphatidylinositol-3-OH kinase, protein kinase C and AMP-activated protein kinase (52).

As high blood glucose levels, insulin resistance and hyperinsulinemia often exist in T2DM patients, these factors would result in abnormal lipid metabolism (53, 54). Hydrogen gas shows a favorable effect in reducing TC level in the present study, showing the same trend as the study of hydrogen treatment before (37, 38, 40). HDL plays a beneficial role in transporting cholesterol reversely (55) which significantly increases in the HI group, but this effect is similar in patients without molecular hydrogen treatment. Previous hydrogen RCTs showed contradictory effects of HDL in metabolic syndrome (32, 42, 56). Although the significant reduction in LDL level presents in both the cohorts, no significant difference is determined between two groups. At the 6-month follow-up period, TG shows a decreasing trend without significance in both groups. It is consistent with previous molecular hydrogen study (52), while hydrogen-water was reported to restrain the increase of plasma TG in another diabetic mice model (57).

Hydrogen gas would result in greater recovery of insulin resistance and β-cell function as HOMA-β and HOMA-IR display a greater amelioration in the HI group. Multiple previous studies showed similar result (37–39, 41). Ectopic deposition of lipids in liver tissue is a non-negligible reason for insulin resistance (58). Hydrogen could suppress this process, which might be a reason for the insulin resistance improvement effect (40).

As ADA declares that the appropriate HbA1c goal for nonpregnant adults is reaching< 7% (53 mmol/mol) (59), Patients treated with HI achieve a 172% higher likelihood of reaching HbA1c< 7% at the follow-up period with an OR (95% CI) of 2.72 (1.50, 4.38) in comparison to the control group in this study.

The gastrointestinal adverse event is the most common AE in both cohorts while no severe adverse event is observed. Compared to the control group, HI therapy is associated with the lower incidence of several AEs including hypoglycemia, vomiting, constipation and giddiness, which indicates that molecular hydrogen treatment would result in fewer side effects and increase safety for human body. In previous studies, hydrogen treatment has been proved to be a safe therapy (60, 61).

The therapeutic mechanism of hydrogen is due to selectively address of OH·, which could damage cellular components and result in cellular necrosis and apoptosis with no targeted detoxification route (17, 24). Previous animal diabetic models proved that molecular hydrogen could improve diabetes and obesity by enhancing the expression of Hepatic fibroblast growth factor 21 (57). Recently, another study revealed that hydrogen may lower blood glucose and lipid *via* restraining TLR4/MyD88/NF-κB signaling (37).

This study has several limitations: First of all, due to its retrospective nature, the evaluation of the therapeutic response of hydrogen treatment might be influenced by inevitable sample bias including but not limited to the variability of concurrent medication, deviation of patient selection and ambiguous metabolism within human body. Furthermore, the AEs are extracted from medical records, leading to the possibility of a few neglected incidents. Lastly, the present study only includes Chinese patients with T2DM, and it is necessary to verify the effects of hydrogen gas in populations from other countries. Despite these limitations, this observational study would provide valuable real-world data which have never been reported previously and present nonnegligible inspiration for existing clinical practice.

## 5 Conclusion

In summary, HI therapy as an adjunct treatment improves the glycemic control of Chinese T2DM patients in a real-world setting, ameliorating the lipid profile and insulin resistance, increasing the likelihood of reaching the HbA1c< 7% target after 6-month treatment with a lower incidence of AE. It reveals an optional and effective strategy for the clinical treatment of T2DM.

## Data availability statement

The raw data supporting the conclusions of this article will be made available by the authors, without undue reservation.

## Ethics statement

The studies involving human participants were reviewed and approved by Ethics Committee of Medical College of Qingdao University. The patients/participants provided their written informed consent to participate in this study.

## Author contributions

ZZ, HJ and TN contributed to the study concept and design. TN contributed to the data collection. ZL and RS performed the statistical analyses and interpreted the results. YL and YZ wrote the initial draft of the manuscript. TN reviewed and edited the manuscript. All authors contributed to the article and approved the submitted version.
